# Apigenin Attenuates Oxidative Injury in ARPE-19 Cells thorough Activation of Nrf2 Pathway

**DOI:** 10.1155/2016/4378461

**Published:** 2016-08-31

**Authors:** Xinrong Xu, Min Li, Weiwei Chen, Haitao Yu, Yan Yang, Li Hang

**Affiliations:** ^1^Department of Ophthalmology, Affiliated Hospital of Nanjing University of Chinese Medicine, Nanjing 210029, China; ^2^School of Pharmacy, Nanjing University of Chinese Medicine, Nanjing 210023, China

## Abstract

The current study was aimed at evaluating the therapeutic implication of apigenin and to elucidate the underlying mechanism. The* tert*-butyl hydroperoxide (*t*-BHP) at 200 *μ*M was used to induce oxidative stress-associated injury in ARPE-19 cells. Apigenin at concentrations less than 800 *μ*M did not cause cytotoxic effects on ARPE-19 cells. Cell viability assay showed that apigenin at 200 *μ*M significantly promoted cell survival in* t*-BHP-treated ARPE-19 cells. Additionally, apigenin at 100 *μ*M significantly protected ARPE-19 cells from* t*-BHP-induced apoptosis. Molecular examinations demonstrated that apigenin at 400 *μ*M significantly upregulated the mRNA and protein expression of Nrf2 and stimulated its nuclear translocation in ARPE-19 cells treated with or without* t*-BHP. Apigenin 400 *μ*M also significantly elevated the expression of HO-1, NQO1, and GCLM at both mRNA and protein levels in the presence or absence of* t*-BHP. Furthermore, apigenin at 400 *μ*M significantly increased the activities of SOD, CAT, GSH-PX, and T-AOC and reduced the levels of ROS and MDA in* t*-BHP-treated ARPE-19 cells. However, these effects of apigenin were all abolished by being transfected with Nrf2 siRNA. Collectively, our current data indicated that apigenin exerted potent antioxidant properties in ARPE-19 cells challenged with* t*-BHP, which were dependent on activation of Nrf2 signaling.

## 1. Introduction

Age-related macular degeneration (AMD) represents the leading cause of blindness in the elderly population, and its prevalence increases tremendously with every decade after the age of 50 years all over the world [1]. The early stage of AMD is featured by alterations of retinal pigmented epithelium (RPE) and subretinal deposits between the RPE and Bruch's membrane [2]. AMD can develop to choroidal neovascularization characterized by new blood vessels that invade the macula, which is a rapidly deteriorating stage of AMD, also known as wet AMD [3]. Therefore, vascular endothelial growth factor (VEGF) as the most important regulator of angiogenic network has been considered as a key target for current treatment of wet AMD, and several VEGF inhibitors such as bevacizumab, pegaptanib, or ranibizumab have demonstrated promising therapeutic benefits in clinical context [4]. In addition, dry AMD can slowly progress to geographic atrophy, which is the late-onset form that results in RPE degeneration in the macula [5]. Unfortunately, there are no available treatments for dry AMD so far.

Mounting evidence indicates that oxidative stress plays a key role in the pathogenesis of dry AMD. Oxidative stress refers to cellular or molecular damage caused by reactive oxygen species (ROS), which especially occurs in age-related conditions as a result of disrupted balance between the ROS production and antioxidant defense response [6]. It has been well-established that the transcription factor Nrf2 (nuclear factor erythroid 2-related factor 2) functions as the master regulator of a highly conserved protective molecular response to oxidative stress in mammal cells. Under basal conditions, Nrf2 physically binds to the negative regulator Keap1 for ubiquitination and proteasomal degradation within the cytoplasm, thus limiting Nrf2 activity. However, under oxidative circumstances, Nrf2 is released from Keap1 and translocates into the nucleus, where it binds to antioxidant response elements (AREs), thus activating transcription of its target genes encoding phase II metabolizing enzymes and antioxidases, including heme oxygenase-1 (HO-1), quinone oxidoreductase-1 (NQO1), glutathione peroxidase (GSH-PX), glutamate-cysteine ligase modifier subunit (GCLM), glutathione, and epoxide hydrolase [7]. There has been evidence that Nrf2-deficient mice developed ocular pathology similar to cardinal features of human AMD and deregulated autophagy is likely a mechanistic link between oxidative injury and inflammation [8]. More understanding of the potential role of Nrf2 in the pathogenesis of dry AMD may provide novel opportunities for therapeutic intervention for the prevention of progression to advanced disease.

Apigenin is a flavonoid compound abundantly present in common fruits, such as grapefruit, and plant-derived beverages and vegetables, such as parsley, onions, oranges, tea, chamomile, and wheat sprouts, and in some seasonings [9]. This compound has been increasingly reported to have various pharmacological activities including anti-inflammation [10], antivirus [11], antioxidation [12], and anticancer [13]. Given the potent antioxidative effects of apigenin, we herein hypothesized that this compound may have therapeutic implication for dry AMD. To this end,* tert*-butyl hydroperoxide (*t*-BHP) was used to establish the oxidative stress-induced cellular model in human RPE cell line ARPE-19, which is isolated from human retinal pigmented epithelium and from stable monolayers exhibiting morphological and functional polarity [14]. ARPE-19 cells have structural and functional properties characteristic of RPE cells* in vivo* and are valuable for* in vitro* studies of RPE physiology. We used this model to examine the protective effects of apigenin and to elucidate the underlying mechanism.

## 2. Materials and Methods

### 2.1. Regents and Antibodies

*t*-BHP and apigenin were purchased from Sigma-Aldrich (Saint Louis, MO, USA). The primary antibodies against Nrf2, HO-1, GCL, NQO1, *β*-actin, and Lamin B and the secondary antibody Goat Anti-Rabbit IgG/HRP used in Western blot analyses were all obtained from Abcam (Cambridge, UK).

### 2.2. Cell Culture

Human RPE cell line ARPE-19 was purchased from the American Type Culture Collection (USA). ARPE-19 cells were cultured in Dulbecco's modified eagle medium (DMEM; Invitrogen, Grand Island, NY, USA) supplemented with 10% fetal bovine serum (FBS; Sijiqing Biological Engineering Materials Co., Ltd., Hangzhou, China), 100 U/mL penicillin, and 100 mg/mL streptomycin and grown in a 95% air and 5% CO_2_ humidified atmosphere at 37°C.

### 2.3. Establishment of Oxidative Injury Model in ARPE-19 Cells

ARPE-19 cells were seeded in 96-well plates (1 × 10^4^/well) and cultured in DMEM with 10% FBS for 24 h. Cells were treated with* t*-BHP at concentrations of 50, 100, 200, 300, 400, and 500 *μ*M for 24 h. Then the medium was replaced with 100 *μ*L phosphate buffered saline containing 0.5 mg/mL 3-(4,5-dimethylthiazol-2-yl)-2,5-diphenyl tetrazolium bromide (MTT) incubating at 37°C for 4 h. Next, dimethylsulfoxide of 200 *μ*L was added to dissolve the crystals. The spectrophotometric absorbance at 490 nm was measured by a SPECTRAmax™ microplate spectrophotometer (Molecular Devices, Sunnyvale, CA, USA). Cell growth inhibition curve was drawn and the half-inhibition concentration (IC_50_) was selected as appropriate concentration of* t*-BHP for establishing oxidative injury model in ARPE-19 cells. Cell growth inhibition rate = (test OD value − blank OD value)/(control OD value − blank OD value) × 100%. Cell survival rate = [1 − (test OD value − blank OD value)/(control OD value − blank OD value)] × 100%. Experiments were performed in triplicate.

### 2.4. Evaluation of Toxicity of Apigenin in ARPE-19 Cells

ARPE-19 cells were seeded in 96-well plates (1 × 10^4^/well) and cultured in DMEM with 10% FBS for 24 h. Cells were treated with apigenin at concentrations of 10, 50, 100, 200, 400, and 800 *μ*M for 24 h. Cell viability was evaluated using MTT assay as described above. Cell survival rate was calculated to evaluate the cellular toxicity of apigenin. Experiments were performed in triplicate.

### 2.5. Evaluation of Apigenin Protection of ARPE-19 Cells against Oxidative Injury

ARPE-19 cells were seeded in 96-well plates (1 × 10^4^/well) and cultured in DMEM with 10% FBS for 24 h. Cells were pretreated with apigenin at concentrations of 100, 200, and 400 *μ*M for 6 h. Then cells were additionally treated with* t*-BHP at 200 *μ*M for 24 h. Cell viability was evaluated using MTT assay as described above to evaluate apigenin protection against oxidative injury in ARPE-19 cells. Experiments were performed in triplicate.

### 2.6. Analyses of Apoptosis

ARPE-19 cells were treated with apigenin at concentrations of 100, 200, and 400 *μ*M for 6 h and then were additionally treated with* t*-BHP at 200 *μ*M for 24 h. Apoptotic rates were determined by flow cytometry using Annexin V-FITC apoptosis assay kits (Nanjing KeyGen Biotech Co., Ltd., Nanjing, China) according to the protocol. Apoptotic cells were defined as the cells situated in the right two quadrants of each plot and the percentages were determined by flow cytometry (FACSCalibur; Becton, Dickinson and Company, Franklin Lakes, NJ, USA). The data were analyzed using the software CELLQuest. Experiments were performed in triplicate.

### 2.7. Cell Transfection with Nrf2 siRNA

Nrf2 siRNA (Shanghai GenePharma Co. Ltd., Shanghai, China) of 100 pmol was added to 100 *μ*L medium without serum and antibiotics and incubated at room temperature for 5 min. The final concentration of Nrf2 siRNA was 100 nM. Lipofectamine 2000 reagent (Invitrogen, USA) of 25 *μ*L was added to 75 *μ*L medium without serum and antibiotics and incubated at room temperature for 5 min. The above two solutions were mixed well at room temperature for 20 min, and about 200 *μ*L transfection complex was obtained. Then the medium of 800 *μ*L without serum and antibiotics was added to the 200 *μ*L transfection complex and mixed well, and the transfection complex solution of 1000 *μ*L was obtained. ARPE-19 cells were incubated with the transfection complex solution at 37°C for 8 h and then were reincubated in complete medium at 37°C for an additional 16 h. Control siRNA (Shanghai GenePharma Co. Ltd., Shanghai, China) is a nontargeting 20–25 nt siRNA designed as a negative control.

### 2.8. Determination of Antioxidant System

ARPE-19 cells were treated with apigenin at 400 *μ*M and/or transfected with Nrf2 siRNA for 6 h and then additionally treated with* t*-BHP at 200 *μ*M for 24 h. Cells were broken up by ultrasound treatment and subjected to centrifugation to obtain the supernatant. The activities of superoxide dismutase (SOD), catalase (CAT), GSH-PX, malondialdehyde (MDA), and the total antioxidant capacity (T-AOC) were determined using corresponding enzyme-linked immunosorbent assay kits (Nanjing Jiancheng Bioengineering Institute, Nanjing, China) according to the instructions of the manufacturer. Experiments were performed in triplicate.

### 2.9. Determination of ROS Level

ARPE-19 cells were treated with apigenin at 400 *μ*M and/or transfected with Nrf2 siRNA for 6 h and then additionally treated with* t*-BHP at 200 *μ*M for 24 h. The fluorescent probe 2′,7′-dichlorodihydrofluorescein diacetate (DCFH-DA) was used to measure the intracellular generation of ROS. ARPE-19 cells were treated with 10 *μ*M DCFH-DA for 20 min at 37°C and then washed three times with medium. Cells were incubated with apigenin at 400 *μ*M and/or transfected with Nrf2 siRNA for 6 h and then additionally treated with* t*-BHP at 200 *μ*M for 24 h. The fluorescence was determined by flow cytometry (FACSCalibur; Becton, Dickinson and Company, Franklin Lakes, NJ, USA) at 488 nm wavelength. Experiments were performed in triplicate.

### 2.10. Real-Time PCR

Total RNA was isolated from treated cells using Trizol reagent (Sigma, St. Louis, MO, USA) following the protocol provided by the manufacturer. Real-time PCR was performed as described previously [15]. *β*-actin was used as the invariant control. Fold changes in the mRNA levels of target genes related to *β*-actin were calculated as suggested by Schmittgen et al. [16]. Experiments were performed in triplicate. The primers of genes (GenScript, Nanjing, China) were as follows:* Nrf2*: (forward) 5′-TGAGGTTTCTTCGGCTACGTT-3′ and (reverse) 5′-CTTCTGTCAGTTTGGCTTCTGG-3′;* HO-1* (forward) 5′-CTGGAGGAGGAGATTGAGCG-3′ and (reverse) 5′-ATGGCTGGTGTGTAGGGGAT-3′;* NQO1* (forward) 5′-GCGTCTGGAGACTGTCTGGG-3′ and (reverse) 5′-CGGCTGGAATGGACTTGC-3′;* GCLM* (forward) 5′-ATCAAACTCTTCATCATCAAC-3′ and (reverse) 5′-GATTAACTCCATCTTCAATAGG-3′; *β-actin* (forward) 5′-CCACACCTTCTACAATGAGC-3′ and (reverse) 5′-GGTCTCAAACATGATCTGGG-3′.

### 2.11. Western Blot Analyses

Whole cell protein extracts were prepared from treated cells with radioimmunoprecipitation assay buffer containing 150 mM NaCl, 50 mM Tris, 0.1% sodium dodecyl sulphate, 1% Nonidet P-40, and 0.5% deoxycholate supplemented with protease inhibitor phenylmethylsulfonyl fluoride. In examining Nrf2 expression, nuclear proteins and cytoplasmic proteins were separated using a Bioepitope Nuclear and Cytoplasmic Extraction Kit (Bioworld Technology, St. Louis Park, MN, USA) according to the protocol. Proteins (50 *μ*g/well) were separated by SDS-polyacrylamide gel, transferred to a PVDF membrane (Millipore, Burlington, MA, USA), and blocked with 5% skim milk in Tris-buffered saline containing 0.1% Tween 20. Target proteins were detected by corresponding primary antibodies and subsequently by horseradish peroxidase-conjugated secondary antibodies. Protein bands were visualized using chemiluminescence reagent (Millipore, Burlington, MA, USA). Equivalent loading was confirmed using an antibody against *β*-actin for total proteins and against Lamin B for nuclear proteins. Representative blots were from three independent experiments. The levels of target protein bands were densitometrically determined using Quantity One 4.4.1. The variation in the density of bands was expressed as fold changes compared to the control in the blot after normalization to *β*-actin or Lamin B.

### 2.12. Statistical Analysis

Data were presented as mean ± SD, and results were analyzed using SPSS16.0 software. The significance of difference was determined by one-way ANOVA with the* post hoc* Dunnett's test. A value of *P* < 0.05 was considered to be statistically significant.

## 3. Results

### 3.1. Apigenin Protects against *t*-BHP-Induced Oxidative Injury in ARPE-19 Cells

**Figure 1:**
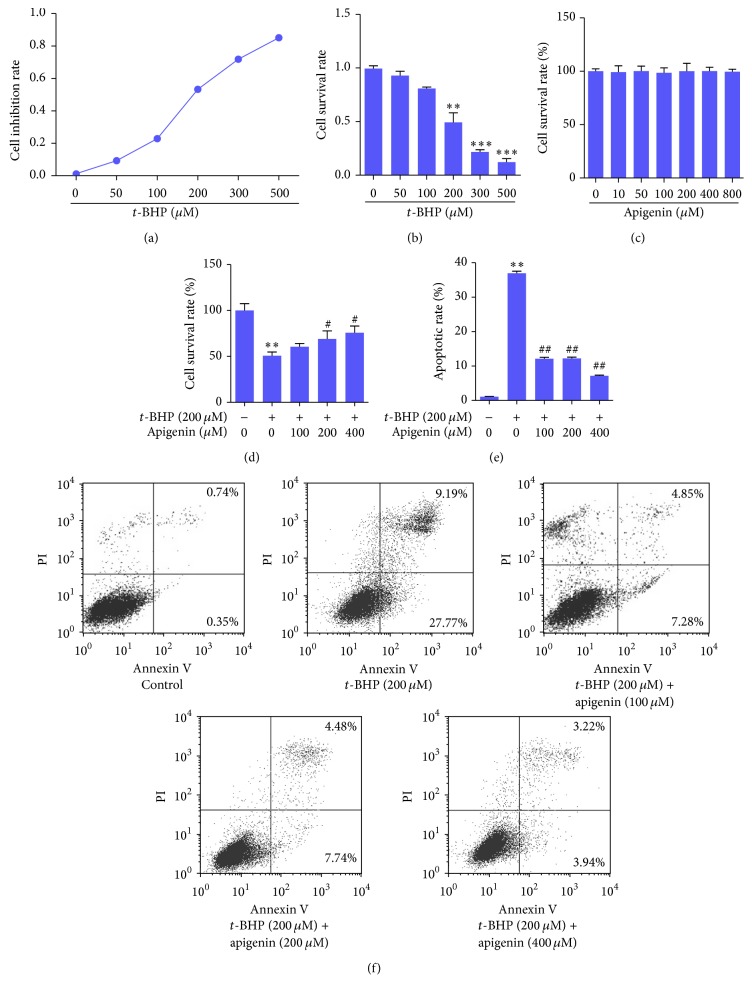
Apigenin protects against* t*-BHP-induced oxidative injury in ARPE-19 cells. (a, b) MTT assay for determining cell inhibition rate and cell survival rate when exposed to* t*-BHP. Significance: ^*∗∗*^ *P* < 0.01 versus control; ^*∗∗∗*^ *P* < 0.001 versus control. (c) MTT assay for determining whether apigenin had toxic effects on cells. (d) MTT assay for determining the protective effects of apigenin on* t*-BHP-treated cells. Significance: ^*∗∗*^ *P* < 0.01 versus control; ^#^ *P* < 0.05 versus* t*-BHP. (e) Flow cytometry analyses of apoptosis using FITC-labeled Annexin V/PI staining. Significance: ^*∗∗*^ *P* < 0.01 versus control; ^##^ *P* < 0.01 versus* t*-BHP. (f) Representative charts of flow cytometry analyses of apoptosis. Cells situated in the right two quadrants of each plot were regarded as apoptotic cells.

In order to establish an oxidative injury model in ARPE-19 cells, we used MTT assay to determine cell growth in the presence of* t*-BHP, a well-known oxidizing agent. The results showed that* t*-BHP increased the cell growth inhibition rate in a concentration-dependent manner (Figure 1(a)) and that* t*-BHP at 200 *μ*M caused a significant reduction in cell survival rate in ARPE-19 cells (Figure 1(b)). Accordingly,* t*-BHP at 200 *μ*M was used to induce oxidative injury in these cells for subsequent experiments. In addition, we found that apigenin at concentrations up to 800 *μ*M did not apparently affect ARPE-19 cell viability, indicating that apigenin might not have toxic effects on these cells (Figure 1(c)). Next, we observed that apigenin concentration dependently increased cell survival rate in ARPE-19 cells exposed to* t*-BHP (Figure 1(d)). Then apoptosis was examined using flow cytometry, and the results demonstrated that ARPE-19 cells exposed to* t*-BHP had significant apoptotic rate compared to control, but apigenin significantly reduced* t*-BHP-induced apoptosis in APRE-19 cells (Figures 1(e) and 1(f)). Taken together, these data collectively indicated that apigenin protected ARPE-19 cells against* t*-BHP-induced oxidative injury.

### 3.2. Apigenin Activates Nrf2 and Its Target Genes Involved in Antioxidant System in ARPE-19 Cells

**Figure 2:**
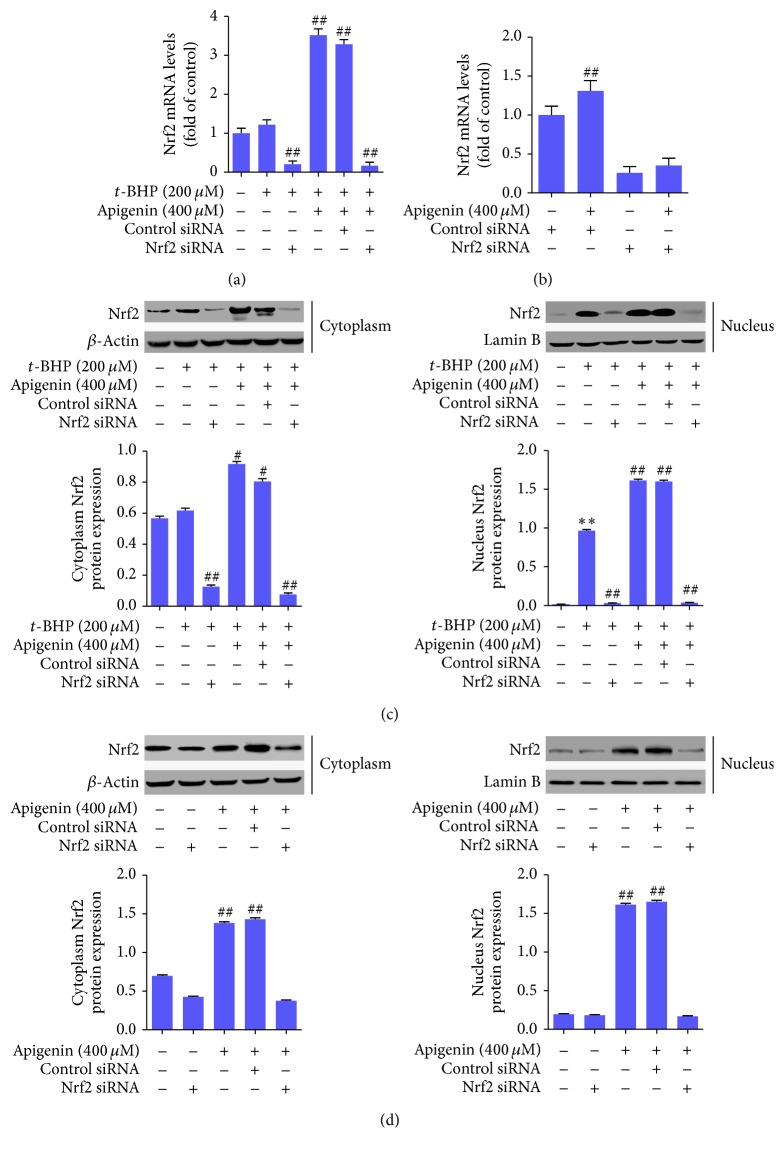
Apigenin upregulates Nrf2 expression and promotes its nuclear translocation in ARPE-19 cells. (a) Real-time PCR analyses of Nrf2 mRNA expression in* t*-BHP-treated ARPE-19 cells. Significance: ^##^ *P* < 0.01 versus* t*-BHP. (b) Real-time PCR analyses of Nrf2 mRNA expression in ARPE-19 cells in the absence of* t*-BHP. Significance: ^##^ *P* < 0.01 versus control siRNA. (c) Western blot analyses of protein abundance of Nrf2 in cytoplasm and nucleus in* t*-BHP-treated ARPE-19 cells with quantification. Significance: ^*∗∗*^ *P* < 0.01 versus control, ^#^ *P* < 0.05 versus* t*-BHP, and ^##^ *P* < 0.01 versus* t*-BHP. (d) Western blot analyses of protein abundance of Nrf2 in cytoplasm and nucleus in ARPE-19 cells in the absence of* t*-BHP with quantification. Significance: ^##^ *P* < 0.01 versus control.

**Figure 3:**
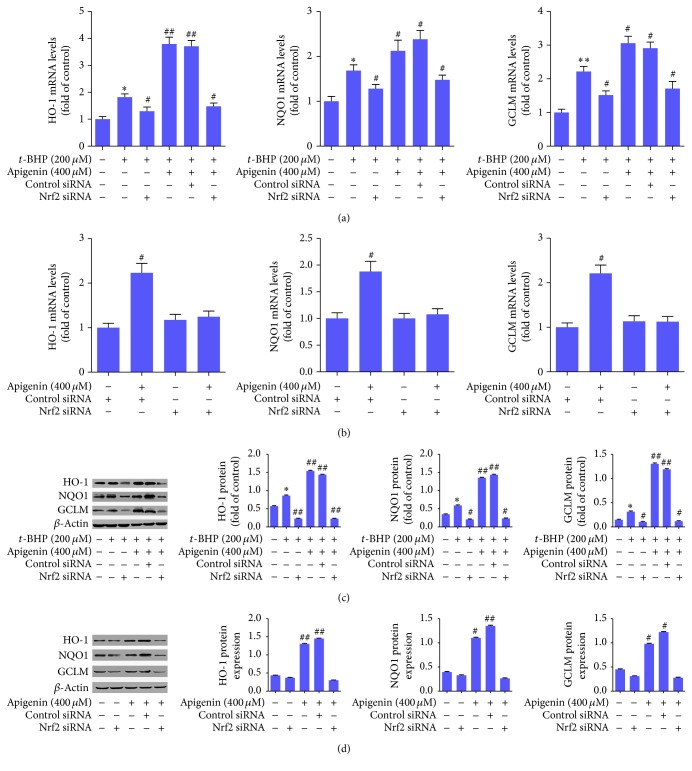
Apigenin increases the expression of Nrf2 target genes involved in antioxidant system in ARPE-19 cells. (a) Real-time PCR analyses of the mRNA expression of HO-1, NQO1, and GCLM in* t*-BHP-treated ARPE-19 cells. Significance: ^*∗*^ *P* < 0.05 versus control, ^*∗∗*^ *P* < 0.01 versus control, ^#^ *P* < 0.05 versus* t*-BHP, and ^##^ *P* < 0.01 versus* t*-BHP. (b) Real-time PCR analyses of the mRNA expression of HO-1, NQO1, and GCLM in ARPE-19 cells in the absence of* t*-BHP. Significance: ^#^ *P* < 0.05 versus control siRNA. (c) Western blot analyses of protein abundance of HO-1, NQO1, and GCLM in* t*-BHP-treated ARPE-19 cells with quantification. Significance: ^*∗*^ *P* < 0.05 versus control, ^#^ *P* < 0.05 versus* t*-BHP, and ^##^ *P* < 0.01 versus* t*-BHP. (d) Western blot analyses of protein abundance of HO-1, NQO1, and GCLM in ARPE-19 cells in the absence of* t*-BHP with quantification. Significance: ^#^ *P* < 0.05 versus control; ^##^ *P* < 0.01 versus control.

We next examined the role of Nrf2 signaling in apigenin protection of ARPE-19 cells using Nrf2 siRNA-mediated knockout approaches. Real-time PCR analyses demonstrated that Nrf2 mRNA was significantly upregulated by apigenin but was significantly decreased by transfection with Nrf2 siRNA in the presence or absence of apigenin in ARPE-19 cells treated with or without* t*-BHP (Figures 2(a) and 2(b)). Consistently, Western blot analyses revealed that the protein abundance of Nrf2 in both cytoplasm and nucleus was significantly increased by apigenin but was significantly reduced by transfection with Nrf2 siRNA in the presence or absence of apigenin in ARPE-19 cells treated with or without* t*-BHP (Figures 2(c) and 2(d)). These data indicated that apigenin could activate Nrf2 signaling in these cells and that the expression and nuclear translocation of Nrf2 could be effectively inhibited by Nrf2 siRNA. Furthermore, we examined the expression of several target genes of Nrf2, which are critically involved in the antioxidant system within cells. The mRNA expressions of HO-1, NQO1, and glutamate-cysteine ligase modifier subunit (GCLM) were all significantly upregulated by* t*-BHP compared to control. Apigenin elevated their mRNA expression significantly compared to the* t*-BHP-treated cells, but transfection with Nrf2 siRNA resulted in reduced mRNA expression of HO-1, NQO1, and GCLM (Figure 3(a)). Apigenin also significantly increased their mRNA expression in ARPE-19 cells in the absence of* t*-BHP (Figure 3(b)). Western blot analyses showed similar consistent results in ARPE-19 cells in the presence or absence of* t*-BHP at the protein level (Figures 3(c) and 3(d)). Collectively, these data suggested that apigenin increased Nrf2 expression and promoted its nuclear translocation, leading to enhanced expression of its target genes implicated in protection against oxidative-caused injuries in ARPE-19 cells.

### 3.3. Activation of Nrf2 Is Required for Apigenin to Exert Antioxidative Properties in *t*-BHP-Treated ARPE-19 Cells

**Figure 4:**
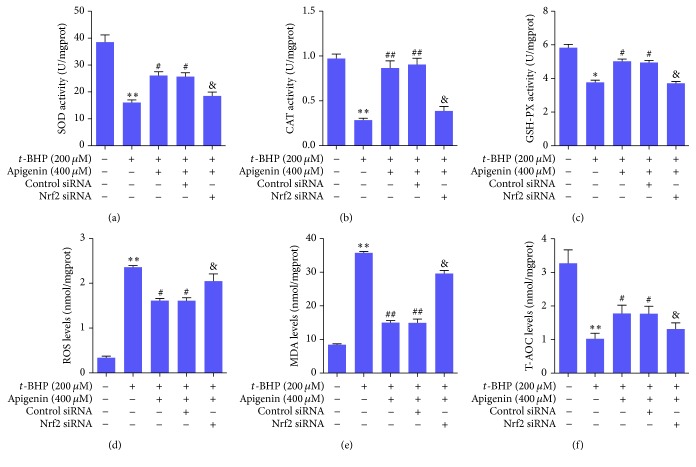
Activation of Nrf2 is required for apigenin to exert antioxidative properties in* t*-BHP-treated ARPE-19 cells. ELISA for SOD activity (a), CAT activity (b), GSH-PX activity (c), ROS levels (d), MDA levels (e), and T-AOC levels (f). Significance: ^*∗*^ *P* < 0.05 versus control, ^*∗∗*^ *P* < 0.01 versus control, ^#^ *P* < 0.05 versus* t*-BHP, ^##^ *P* < 0.01 versus* t*-BHP, and ^&^ *P* < 0.05 versus* t*-BHP + apigenin + control siRNA.

We next evaluated the antioxidative effects of apigenin and their associations with activation of Nrf2 signaling. We measured the activities of a series of phase II metabolizing enzymes and antioxidases in* t*-BHP-treated ARPE-19 cells. The results showed that the enzyme activities of SOD, CAT, and GSH-PX were significantly reduced in* t*-BHP-treated ARPE-19 cells, but apigenin at 400 *μ*M significantly restored the activities of these enzymes. However, the effects of apigenin were significantly abrogated by transfection with Nrf2 siRNA in ARPE-19 cells exposed to* t*-BHP (Figures 4(a)–4(c)). Moreover, the levels of ROS, MDA, and T-AOC, three parameters indicative of oxidative status within cells, were determined. ARPE-19 cells challenged with* t*-BHP exhibited significantly elevated ROS and MDA levels and reduced T-AOC levels. However, apigenin significantly reduced ROS and MDA levels and enhanced T-AOC levels in* t*-BHP-treated ARPE-19 cells, but these effects were apparently abolished by transfection with Nrf2 siRNA (Figures 4(d)–4(f)). Altogether, these results suggested that activation of Nrf2 was required for apigenin to exert antioxidative properties in* t*-BHP-treated ARPE-19 cells.

## 4. Discussion

Effective therapeutic approaches for dry AMD are urgently needed in current clinical context. Increasing evidence from basic and clinical studies highlights that oxidative injury is critically implicated in the pathogenesis of dry AMD, and thus agents with antioxidative properties may be promising therapeutic options for dry AMD [17]. For example, an Age-Related Eye Disease Study (AREDS) evaluated the effects of several antioxidant agents at pharmacological doses including *β*-carotene, vitamin C, vitamin E, zinc, and copper on the progression of AMD and visual acuity, demonstrating that administration of antioxidants could result in 25% risk reduction in advanced AMD progression and 19% risk reduction in moderate vision loss within 5 years [18]. In addition, a recent investigation evaluated the safety and efficacy of OT-551, a disubstituted hydroxylamine with antioxidant properties, for the treatment of geographic atrophy, the advanced atrophic form of AMD, and demonstrated that this agent could be beneficial for maintaining visual acuity in AMD patients [19]. In the present study, we demonstrated that the flavonoid compound apigenin was safe for ARPE-19 cells and significantly increased the survival rate and reduced the apoptotic rate in these cells with oxidative injury caused by* t*-BHP. Apigenin at 400 *μ*M, especially, could provide favorable protective effects on ARPE-19 cells against oxidative injury. Our discoveries suggested apigenin as therapeutic candidate for AMD. Moreover, in view of the fact that oxidative stress is most likely an early (and enduring) toxic stimulus, we assumed that apigenin could also serve as a preventing agent for AMD. Given that flavonoids are well-known for their antioxidant properties, we therefore concentrated on the signaling pathways that regulate intracellular redox system in hope of elucidating the mechanisms of apigenin implicated in treatment for AMD.

The transcription factor Nrf2 is widely acknowledged as a key regulator of antioxidative pathways in mammal cells. A large number of studies have established an important link of Nrf2 signaling to the pathogenesis of AMD. Studies showed that Nrf2-deficient mice exhibited remarkable AMD-like retina alterations [8] and that activation of Nrf2 pathway by pharmacological agents could protect ARPE cells against oxidative-caused damage [20, 21]. Our current data demonstrated that* t*-BHP at 200 *μ*M did not apparently alter the transcription of Nrf2 but significantly elevated the nuclear protein abundance of Nrf2 in ARPE cells. Apigenin also potently increased the transcription of nuclear translocation of Nrf2 and upregulated the expression of a series of phase II metabolizing enzymes in* t*-BHP-treated ARPE cells. These data suggested that Nrf2 nuclear translocation occurred in ARPE cells upon oxidative stimulation, leading to the transcription and expression of several phase II metabolizing enzymes, and that apigenin could potently activate Nrf2 signaling in these cells. Consistently, it was reported that apigenin could restore the silenced status of Nrf2 in skin epidermal JB6 P + cells by CpG demethylation coupled with attenuated activities of DNA methyltransferase and histone deacetylases [22]. Apigenin was also found to significantly activate the Nrf2-antioxidant response element-mediated gene expression and induce anti-inflammatory activities in HepG2 cells [23].

We further demonstrated that apigenin had potent antioxidant properties in ARPE cells, because the activities of antioxidant enzymes SOD, CAT, and GSH-PX and the T-AOC levels were significantly enhanced, but the levels of ROS and MDA were significantly reduced by apigenin. Consistently, the antioxidant effects of apigenin were also demonstrated in many other circumstances. For example, the flavonoid constituents of* Sida cordata*, which are full of apigenin, increased the activity of hepatic antioxidant enzymes including CAT, SOD, POD, GST, GSR, and GSH-Px in rats intoxicated with carbon tetrachloride [24]. The yam peel extracts, in which apigenin is one of the predominant components, could protect rats from liver damage by enhancing the activities of antioxidant enzymes and anti-inflammatory capacity [25]. Our current studies recaptured these results in ARPR-19 cells under oxidative stress, which could be implicated in AMD treatment. Interestingly, we observed that the antioxidant properties of apigenin were dependent on activation of Nrf2 in ARPE-19 cells exposed to* t*-BHP, because loss-of-function approaches with siRNA-mediated Nrf2 knockdown significantly abrogated apigenin upregulation of SOD, CAT, GSH-PX, and T-AOC activities and rescued apigenin downregulation of ROS and MAD levels. These findings indicated that Nrf2 could be a key target molecule for pharmaceutical modulators to protect human retinal epithelial cells against oxidative injury and thus play a beneficial role for AMD treatment. In line with our current data, apigenin was reported to increase Nrf2 expression or activate its function in differentiated PC12 cells [26] and rat primary hepatocytes [27]. However, contrast results also reported that apigenin dramatically reduced Nrf2 expression at both the mRNA and protein levels through downregulation of PI3 K/Akt pathway, leading to a reduction of Nrf2-downstream genes in BEL-7402 cells [28]. Taking these findings together, it was presumed that apigenin regulation of Nrf2 could be dependent on different cell types or pathophysiological contexts. In view of this, we are performing animal studies using Nrf2 knockout mice to validate the apigenin regulation of Nrf2 signaling and the therapeutic effects of apigenin on AMD* in vivo*.

In summary, apigenin exhibited potent protective effects on ARPE-19 cells against* t*-BHP-induced oxidative injury, which were associated with its antioxidant effects dependent on activation of Nrf2 signaling. Our current study strongly indicated that apigenin could be a safe therapeutic option for treatment of dry AMD.
